# Recent Advances in the Utilization of Cellulose from Food Processing Byproducts for the Generation of Aerogels

**DOI:** 10.3390/gels11050359

**Published:** 2025-05-14

**Authors:** Jaspreet Kaur, Ali Ubeyitogullari

**Affiliations:** 1 Department of Food Science, University of Arkansas, Fayetteville, AR 72704, USA; jk041@uark.edu; 2 Department of Biological and Agricultural Engineering, University of Arkansas, Fayetteville, AR 72701, USA

**Keywords:** cellulose, aerogels, supercritical carbon dioxide, nanoporous, circular bioeconomy

## Abstract

Aerogels have garnered significant attention from the scientific community due to their extraordinary properties, including low density, high porosity, low thermal conductivity, and large surface area. These properties make them interesting candidates for diverse applications such as thermal insulation, drug delivery, catalysis, fillers, tissue engineering, and biosensors. However, the production of conventional aerogels is often constrained by environmental issues, the high cost of raw materials, and energy-intensive fabrication methods. In contrast, cellulose aerogels have emerged as promising sustainable materials with the potential to transform various low-cost waste products into high-value biomaterials. Food-processing byproducts provide numerous untapped opportunities for the generation of aerogels. This review highlights the recent advancements in the development of cellulose aerogels derived from food processing byproducts, emphasizing their role in contributing to the circular bioeconomy. Specifically, this study focuses on the fabrication processes of cellulose aerogels from food processing byproducts, which would otherwise go to waste. The review discusses the extraction, gel formation, drying, and functionalization processes for cellulose aerogel formation, along with the environmental and economic benefits of utilizing these waste streams.

## 1. Introduction

**Figure 1:**
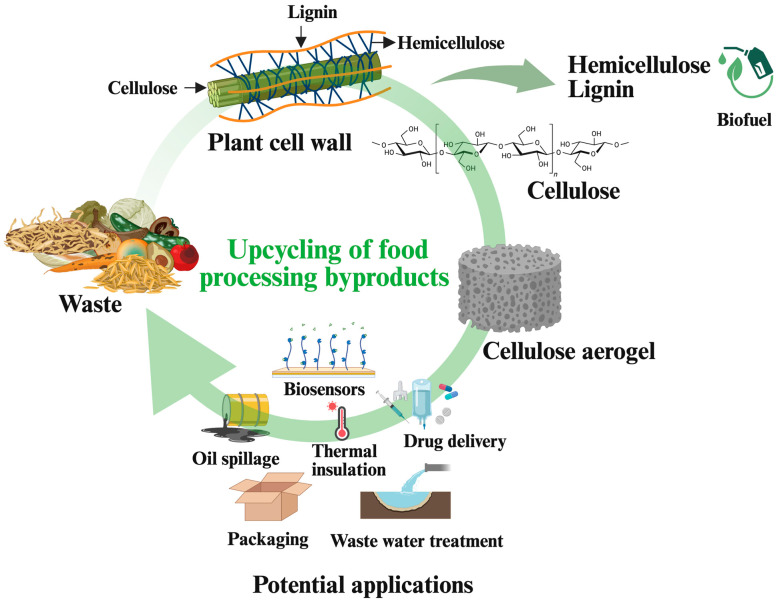
Upcycling of food processing byproducts into cellulose aerogels.

The scarcity of fossil resources and growing environmental concerns have prompted researchers, corporations, and governments to seek cost-effective, biodegradable, and non-toxic biopolymers as sustainable alternatives [1]. Aerogels have emerged as a valuable class of materials to replace those derived from petroleum-based polymers. Among various polymers, cellulose, owing to its biocompatibility, biodegradability, renewability, and non-toxicity, has emerged as a promising biopolymer for generating aerogels [2]. Cellulose aerogels, in addition to the inherent properties of cellulose, exhibit an ultralight structure, high degree of porosity, extensive specific surface area, etc., making them ideal candidates for diverse applications, such as adsorption, catalysis, sound insulation, drug delivery, aerospace materials, and food packaging [1,3,4,5]. Cellulose aerogels are prepared from various sources, including lignocellulosic biomass, bacteria, and marine [1,6]. Food-processing byproducts, such as hemp stems, apple pomace, and spent ground coffee, have emerged as low-cost and sustainable materials to produce cellulose aerogels, contributing to waste valorization and the circular bioeconomy (Figure 1). Upcycling these byproducts into aerogels reduces environmental pollution and generates economic benefits [7,8]. Even though there have been review papers on aerogels from various biopolymers, including starch, chitosan, pectin, and β-glucan [9,10,11], the number of reviews on cellulose aerogels from food processing byproducts is scarce. Therefore, this review explores the synthesis of cellulose aerogels, with a specific focus on their production from food processing byproducts, their key properties, and potential applications. This study can open new avenues for food processing byproducts by upcycling them into high-value products.

## 2. Aerogels

Aerogels are one of the promising sustainable materials with the potential to address current challenges related to resource scarcity and environmental sustainability. According to the International Union of Pure and Applied Chemistry (IUPAC), “Aerogels are non-fluid networks composed of interconnected colloidal particles as dispersed phase in a gas (typically air)” [12]. These are highly porous and lightweight materials with exceptional physicochemical properties, including low thermal conductivity (as low as 0.015 W m^−1^ K^−1^), low density (as low as 1 kg m^−3^), and high specific surface area (as high as 1000 m^2^ g^−1^) [12,13,14]. They also demonstrate exceptional mass transfer capabilities, attributed to their coherent porous solid structure with porosity greater than 95% and pore diameter within 2 to 50 nm [12]. However, the precise definition of aerogels is ambiguous as their physicochemical properties can vary significantly depending on the fabrication process and the material [12,15], where supercritical carbon dioxide (SC-CO_2_) drying is considered the best drying technique to preserve the structure of gels [11,16,17]. Due to their unique physicochemical properties, aerogels are used in diverse fields, including optoelectronics, sound insulation, medical materials, adsorption catalysis, and aerospace materials [10,18]. Moreover, the tendency of various organic and inorganic materials to form composites leads to the development of aerogels, expanding their potential applications [18].

The groundbreaking discovery of aerogels was made in 1931 by Steven Kistler. Silica gel was used as the precursor material, and its corresponding aerogel was prepared via supercritical drying method that involved increasing the temperature and applying pressure beyond the critical point of the gel solvent [12,18,19]. Additionally, he signed a licensing agreement with the Monsanto Corporation in the early 1940s to produce silica aerogel, resulting in the lasting commercial success of his products. By 1942, the aerogel was marketed under the Santocel brand by Monsanto [18].

The market for aerogels has a promising growth potential due to their potential diverse applications [20]. In 2024, the market size of aerogels was valued at USD 0.9 billion and is forecasted to grow at a CAGR of 12.8%, reaching approximately USD 1.6 billion by 2029 [21]. A few commercial producers of aerogels are located worldwide, including in North America (TAASI, Marketech International Inc., CDT Systems Inc., Dow Corning, Cabot Corp., Aerogel Technologies, Nanopore Inc., American Aerogel, Aspen Aerogel), Europe (Active Aerogels, Green Earth Aerogels, Enersens SAS, Keey Aerogels, Svenska Aerogels, Airglass, BASF SE), and Asia (JIOS Aerogel Corp, Tiem Factory Inc., NanoHigh-Tech Co., Ltd.). These commercial suppliers mainly produce silica aerogels, while some produce organic and carbon aerogels [22]. However, the aerogel market faces challenges due to high production costs and expensive raw materials. Researchers are actively focusing on alleviating production expenses and developing more appealing aerogel products for consumers [20].

Aerogels are mainly classified into organic and inorganic aerogels, depending on the precursor used for their preparation [12,23]. Inorganic aerogels are the earliest and most common type, exhibiting porous 3D networks with material-specific properties [18]. Inorganic aerogels are primarily derived from silica, titanium, graphene, etc., and are used for non-food applications [12,24]. Silica-based aerogels have been mostly explored due to their high porosity, low thermal conductivity, and transparency [24]. However, they are brittle, thus offering limited resistance to compression [7,23]. Moreover, the high synthesis cost of silica aerogels further restricts their commercial applications [7,25]. On the other hand, organic aerogels have gained increasing attention due to their biodegradability, non-toxicity, and less environmental impact, leading to a shift in focus towards their production [26]. Organic aerogels can either be carbon-based or polymer-based (synthetic and biopolymers) aerogels [12,23]. Carbon aerogels, derived from biological sources through pyrolysis or combustion, display a porous 3D network and are primarily used as adsorbents and electrodes in electrical devices [27]. Synthetic polymers used to prepare aerogels include melamine, resorcin, polyvinyl alcohol (PVA), etc., and biopolymers include carbohydrates (e.g., polysaccharides) and proteins [12,23]. Polysaccharide-based aerogels are gaining attention due to their potential to replace petroleum-based materials in various industrial processes, promoting a circular bioeconomy. These polysaccharides, including cellulose, starch, alginate, and chitosan, can be derived from plants, bacteria, or algae [28,29]. Due to their biological origin, biodegradability, and the ability to be produced in large quantities at low cost through industrial processes, like biorefineries, polysaccharide-based aerogels are becoming a key area of research [14]. Among the polysaccharides, cellulose, the most abundant biopolymer on Earth, is an interesting raw material for aerogel preparation and can be sourced from plants, marine animals, or bacteria [30]. Cellulose aerogels have gained interest due to various advantages, such as biocompatibility, low-cost biomass source, biodegradability, strong renewability, and high compressive strength [31]. They have emerged as multi-functional materials with applications in thermal insulation, biomedical materials, adsorption, and many other areas [1].

## 3. Cellulose Aerogels

### 3.1. Overview of Cellulose and Its Extraction Process

Cellulose (C_6_H_10_O_5_)_n_ is a natural, unbranched biopolymer, composed of repeating D-glucose units, with β (1 → 4) glycosidic linkage. It is the most abundant polysaccharide on Earth, primarily found in plant cell walls as microfibrils, as well as in algae, and marine species, and it is also synthesized by bacteria [32,33]. Cellulose is most commonly found in its purest form in cotton, comprising 90%, and in wood, making up 40% to 50% [33]. Cellulose was first identified by Anselme Payen, a French chemist, in 1838 and was named “les cellules” (cellulose). Cellulose was identified as a resistant fibrous residue from timber plant tissues treated with acids and ammonia, which was later converted to dextrose using sulfuric acid. He discovered its chemical similarity to sugar and starch, performed chemical analysis, and determined its chemical formula. In 1920, Hermann Staudinger, a German scientist, determined its polymeric structure, consisting of glucose units linked by covalent bonds [33,34]. Cellulose displays unique features due to its structure, including chirality, degradability, and porosity. Moreover, the abundance of hydroxyl groups in cellulose forms a dense hydrogen bond network, enabling both intra- and intermolecular interactions. These hydroxyl groups contribute to its hydrophilicity and allow for various chemical modifications on its surface. Cellulose fibers are composed of hierarchical units, with elementary fibrils that aggregate into microfibrils and eventually form fibers. Cellulose comprises highly ordered crystalline and disordered amorphous regions [32,33,34,35]. Due to the unique physicochemical properties of cellulose, it has versatile applications in many industries, including food, paper and pulp, textile, pharmaceutical, cosmetic, and biomedical [32,36,37].

Lignocellulosic biomass is the primary source for cellulose extraction, consisting of various components such as cellulose, hemicellulose, lignin, pectin, proteins, and extractives. The constituents of the components vary depending on the biomass source, typically comprising 40–60% cellulose, 20–40% hemicellulose, and 10–24% lignin [38,39]. Lignocellulosic biomass includes woody biomass, such as hardwood and softwood, and non-woody biomass like cotton, hemp, kenaf, jute, and flax [40,41]. Agricultural crop residues, including wheat, maize, and rice straws, as well as husk, bagasse, and agroforestry waste, are also significant sources of cellulose [40,42]. Additionally, cellulose can be obtained from municipal and industrial waste, including paper, textiles, yard waste, cardboard, and food scraps [40,43]. Other lignocellulosic sources include food byproducts like pomace from fruit juice and wine production and spent grains from the beer and whiskey industries [40]. Table 1 shows the composition of lignocellulosic components, i.e., cellulose, hemicellulose, and lignin, in some typical food processing byproducts.

**Figure 2:**
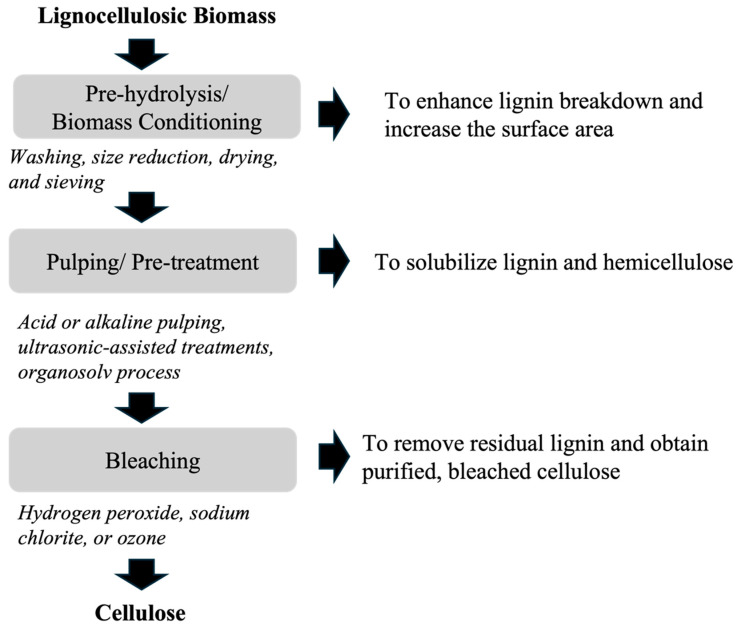
Key steps in cellulose extraction from lignocellulosic biomass.

Cellulose extraction from lignocellulosic biomass involves the separation of cellulose from the biomass, where it is tightly bound to lignin and hemicellulose. The isolation of cellulose fiber involves three main steps that include pre-hydrolysis or biomass conditioning, pulping or pretreatment, and bleaching. Figure 2 shows the schematic of isolating purified cellulose pulp from lignocellulosic biomass [40,51,52]. Biomass conditioning involves washing, size reduction, drying, and sieving, which enhance lignin breakdown and increase the surface area for improved chemical, microbial, and enzymatic reactions in subsequent processes [40,52]. In this step, washing could be performed manually or mechanically, which is followed by chopping to approximately ~2 cm and soaking in deionized water to loosen lignin and eliminate water-soluble components. Subsequently, the material is dried, ground to a fine powder, and screened to separate the particles of specific size [52]. Biomass conditioning is further followed by pulping or pretreatment to solubilize lignin and hemicellulose to achieve cellulose, where the biomass undergoes treatment methods such as acid or alkaline pulping, ultrasonic-assisted treatments, Organosolv process, Deep Eutectic Solvents (DES), or a combination of one or more methods [51]. Alkaline pretreatment is commonly conducted at ambient temperature and pressure, utilizing alkaline reagents, like sodium, potassium, calcium, and ammonium hydroxides to dissolve lignin and hemicellulose, with sodium hydroxide (NaOH) being the most effective [40,51,53]. NaOH is often combined with other chemicals to enhance pretreatment efficiency [40,51,53]. The hydroxide ion of sodium hydroxide breaks down the intermolecular ferulic acid linkage between lignin and hemicellulose fractions [54]. This induces cellulose swelling, reducing its crystallinity, and degree of polymerization, thereby increasing its internal surface area and enzyme accessibility [53,54]. Additionally, kraft pulping is a dominant alkaline pulping process in the paper and pulp industry that utilizes high temperatures (145–170 °C) and pressure to treat woody biomass in a solution of sodium hydroxide and sodium sulfide, known as white liquor [55]. The final step involves bleaching using bleaching agents such as hydrogen peroxide (H_2_O_2_), sodium chlorite (NaClO_2_), or ozone (O_3_) to remove residual lignin and obtain purified, bleached cellulose [51]. Usually, bleaching involves heating alkali-treated fibers in a sodium chlorite solution under acidic conditions, created using NaOH and acetic acid buffer, generating chlorine dioxide (ClO_2_) for lignin removal. The use of H_2_O_2_ enhances the breakdown of lignin-hemicellulose linkages. Multiple bleaching cycles are performed to ensure complete lignin removal, resulting in white fibers, indicating successful process completion [56,57]. The bleaching process can significantly impact the physical properties of fiber, such as its strength and durability [57].

The purified cellulose can be chemically modified and processed to produce valuable products, such as bioplastics. It can be shaped into diverse forms, including cellulose hydrogels, aerogels, membranes, and fibrous materials, unlocking its full potential for various applications [58].

### 3.2. Properties and Classification of Cellulose Aerogels

Cellulose aerogels are highly porous materials (84.0–99.9% porosity) with a three-dimensional interconnected network, derived from cellulose. They are ultra lightweight with densities ranging from 0.0005 to 0.35 g∙cm^−3^ and exhibit extensive specific surface area (10–975 m^2^/g), superior compressive strength (5.2 kPa–16.67 MPa), and hydrophilicity. Additionally, they offer the key advantages of renewability, biodegradability, biocompatibility, abundant reserves, and low cost [1,2,59]. Cellulose aerogels were first produced from viscose by Ookuna’s group in 1993 and were referred to as “porous cellulose”. This discovery paved the way for the rapid development of cellulose aerogels, which have gained significant attention for their widespread applications [59].

Cellulose aerogels can be broadly classified into four categories: bacterial cellulose aerogels, nanocellulose aerogels, cellulose derivative aerogels, and regenerated cellulose aerogels. This classification is based on the molecular structure, chemical composition, and synthesis techniques [1,60]. Bacterial cellulose aerogels are derived from bacterial cellulose obtained from static bacterial cultures. They are prepared by removing bacteria and other impurities, followed by drying. Bacterial cellulose aerogels offer the benefits of high purity as they lack lignin, hemicellulose, and other organic impurities. Apart from this, they are highly crystalline with a high polymerization degree. However, they are limited by long processing time, significantly low yield, and high cost [1,61]. Cellulose derivative aerogels are aerogels synthesized from chemically modified cellulose, such as carboxymethyl cellulose, hydroxypropyl methylcellulose, triacetyl cellulose, and cellulose acetate. The substitution of hydroxyl groups with functional groups reduces their ability to form hydrogen bonding, thereby necessitating the use of crosslinking agents for successful gelation [1,60]. Furthermore, in the third category, nanocellulose aerogels are derived from nanocellulose fibers and prepared by dispersing nanocellulose in water via ultrasonic or mechanical methods, followed by drying [1,60,62]. They exhibit low shrinkage (<7%) and a high compressive modulus (up to 5.93 MPa) due to their high crystallinity and aspect ratio [1,60]. However, it possesses low optical transparency, limited elasticity, and high production costs due to high energy and chemical usage [1]. Regenerated aerogels are gaining significant attention due to their simple and cost-effective production process. Cellulose I (e.g., cotton) is converted into the more thermodynamically stable cellulose II (regenerated cellulose) via dissolution or mercerization treatment [1,59]. Further, the preparation of regenerated cellulose aerogels involves a series of steps, including cellulose dissolution, cellulose regeneration, solvent exchange, and drying. However, the process is time-consuming, and the shrinkage rate exceeds 30% [1,60]. Additionally, cellulose aerogels can also be classified based on their functional performance, such as adsorption, thermal insulation, or biomedical, offering the synthesis of aerogels based on specific applications.

### 3.3. Synthesis of Cellulose Aerogels

The fabrication of cellulose aerogels is similar to the traditional method of producing organic or inorganic aerogels. Typically, the cellulose aerogels are produced by following gel formation, solvent exchange, and drying steps. Firstly, depending on the source, cellulosic fibers are extracted, and it mainly involves the delignification step. This step improves the accessibility of cellulose for transforming it into aerogels [63,64]. The extraction process of cellulosic fibers from lignocellulosic biomass is discussed in Section 3.1. After the extraction of cellulose, cellulose is dissolved or dispersed in a dispersant to break down the intra- and intermolecular hydrogen network of cellulose. This might also involve an additional step of reducing the particle size of cellulose by converting it into nanocellulose, i.e., cellulose nanocrystals (CNCs) and cellulose nanofibers (CNFs). To dissolve cellulose, various types of solvents can be used, such as aqueous complexing agents (cuprammonium hydroxide, cadmium hydroxide in aqueous ethylenediamine solution, iron tartrate in alkaline aqueous solution), alkaline systems (aqueous sodium hydroxide, lithium hydroxide) and nonaqueous systems (urea, thiourea, zinc oxide, N-methylmorpholine-N-oxide, and ionic liquids) [5]. Additionally, cellulose derivatives, produced by chemical modification of cellulose, can easily dissolve in water or specific organic solvents, such as carboxymethyl cellulose (CMC) and hydroxypropyl methylcellulose (HPMC) in water; triacetyl cellulose (TAC) in dioxane/isopropanol; ethyl cellulose (EC) in dichloromethane; and cellulose acetate (CA) in acetone, dichloromethane, dimethylacetamide, and dimethylformamide [1,5]. After dissolution, the liquid phase is transformed into a solid-gel phase via the sol–gel method, and this governs the formation of a 3D porous network structure in cellulose aerogels [1,5,65]. The formation of a gel from a cellulose suspension is fundamentally driven by polymer agglomeration or phase separation, occurring through physical and chemical crosslinking. Physical crosslinking relies on Van der Waals forces, hydrogen bonding, electrostatic interactions, and chain entanglement, whereas chemical crosslinking involves the formation of covalent bonds using agents like epichlorohydrin (ECH) and N, N-methylene bisacrylamide (MBA), creating a stable 3D network [1,5,64]. The rate of gelation depends on the concentration of cellulose solution and the temperature [1,5].

Once the cellulose gels are formed, they undergo a solvent exchange step, which generally aims to replace the water in the system (in the case of hydrogels) with a solvent that has higher solubility/volatility. This step helps protect the 3D network structure and shorten the drying time [2,6]. If the solvent exchange is conducted using ethanol, the produced gels are called alcogels. The solvent exchange is conducted using multiple steps, where the concentration of the solvent is gradually increased from 0 to 100% (e.g., 30, 50, 70, and 100%). This gradual increase in the solvent concentration minimizes shrinkage in the samples [2].

Further, the synthesis is followed by drying, a crucial step for cellulose aerogel preparation that involves the removal of liquid from the gel. The choice of drying method significantly impacts the morphology of cellulose aerogels. The conventional drying process generally causes the gel pore structure to collapse and crack due to the capillary pressure induced by air–liquid interface bending [1,5,11]. Supercritical drying and freeze-drying are commonly used methods for drying the formed hydrogels [1,5]. However, in this study, mainly supercritical drying studies are covered, and supercritical drying is generally referred to as generating aerogels compared to the cryogels generated by freeze-drying. The supercritical drying process involves the removal of the liquid–vapor meniscus for the formation of cellulose aerogels. In this method, the wet gel, after solvent exchange, is placed in a high-pressure heating chamber, where temperature and pressure are increased until the solvent reaches its critical point. After reaching the supercritical point, the solvent eliminates the liquid–vapor interface, preventing the formation of surface tension and preserving the 3D-porous structure of the gel. SC-CO_2_ drying is the most commonly used method due to the relatively mild critical point (31.1 °C, 7.4 MPa), low cost, and non-toxicity of CO_2_. During the drying process, SC-CO_2_ diffuses into the gel network and dissolves the solvent, causing the liquid to expand and spill out of the gel network. As the SC-CO_2_ fills the gel pores, supercritical conditions eliminate an intermediate vapor–liquid transition. The absence of surface tension prevents pore collapse damage and ensures the integrity of the gel structure [5,6,63].

Overall, the synthesis of cellulose aerogels is governed by several key parameters, including the type and concentration of precursor, surfactants, catalyst type and concentration, pH, temperature, and drying methods. These factors are responsible for density, porosity, molecular interconnectivity, gelation kinetics, and crosslinking density, which are crucial while synthesizing aerogels [66]. The performance of the synthesized aerogels is based on several indicators, which involve structural (e.g., pore size and volume, surface area), mechanical (e.g., compressive strength and elasticity), thermal (e.g., thermal conductivity), and functional indicators (e.g., adsorption capacity) [1].

### 3.4. Factors Influencing Properties and Structure of Cellulose Aerogels

The properties and structure of cellulose aerogels are significantly influenced by various factors, including the choice of dissolution solvent, gel formation, and drying method. The choice of solvent system for dissolution dictates the arrangement of cellulose chains, thus impacting aerogel’s morphology [6,67]. Similarly, the addition of additives, such as oil droplets and surfactants, before gel formation and the use of antisolvents during cellulose regeneration and drying can further tailor the porosity and structural characteristics of the aerogel. Different drying methods, like SC-CO_2_ drying and slow or fast freezing lyophilization, produce materials with different porous networks [67]. For instance, robust freezing generates isotropic porous networks, whereas directional ice-templating exhibits honeycomb-like 2D pores along the freezing direction [6]. In addition, SC-CO_2_ drying has been shown to result in aerogels with higher surface areas compared to the cryogels obtained via freeze-drying. In a previous study, lupin hull cellulose aerogels (115 m^2^/g) showed significantly higher surface areas than cryogels (20 m^2^/g). This is mainly due to the ice crystal formation during freezing, which negatively impacted the distribution of cellulose fibers, resulting in a lower surface area [62]. Therefore, SC-CO_2_ drying is usually preferred over freeze drying as it leads to the formation of aerogels with high pore volume, high porosity, and high specific surface area compared to freeze drying. Moreover, the aerogel structure is preserved via SC-CO_2_ drying due to gas–liquid properties of SC-CO_2_, whereas freeze drying can damage nanostructured gels as it promotes ice crystal growth and internal stress within the pores, leading to matrix fracture [68,69,70]. Additionally, aerogels formed from SC-CO_2_ drying have a high bioactive loading capacity compared to freeze drying, further supporting the preference of SC-CO_2_ drying over freeze drying in applications such as drug delivery [69].

Additionally, the molecular weight of cellulose has also shown a significant impact on the properties of aerogels. A study reported higher surface areas (197 m^2^/g vs. 85 m^2^/g), denser structure, and finer nanofibrils with enhanced thermal stability of aerogels using high molecular weight cellulose fibers compared to those prepared with low molecular weight cellulose [71]. The self-association behavior of cellulose derivatives has also been reported to impact the formation of aerogels, as depicted by stronger and more resilient aerogel structures formed by carboxymethyl cellulose (CMC) compared to non-functionalized microcrystalline cellulose (MCC) [72]. This suggests that various factors can be tuned to synthesize cellulose aerogels to meet specific application requirements. A comprehensive understanding of the relationships among these variables is crucial for optimizing the synthesis process of cellulose aerogels and achieving the desired structural and functional properties.

The physicochemical properties and structure of cellulose aerogels are intrinsically linked to the performance of cellulose aerogels in various applications. For instance, low thermal conductivity and high pore volume of cellulose aerogels are suitable for thermal insulation applications, such as refrigerator insulation materials [73]. Additionally, high surface area, low density, and excellent water absorption capacity of cellulose aerogels are advantageous for water purification and wastewater treatment, as they provide numerous active sites for efficient contaminant uptake [74,75]. Likewise, high mechanical strength and low density are key determinants in food packaging applications [4]. Consequently, the aerogel structure can be tailored by tuning various factors, such as the solvent system and drying method, to meet the requirements of targeted applications.

## 4. Cellulose Aerogels from Food Processing Byproducts

While cellulose aerogels can be synthesized from various sources, food processing byproducts are increasingly recognized as valuable raw materials for their role in reducing food waste, enhancing sustainability, and promoting the circular economy. A significant amount of food waste is generated each year, including hulls, husks, peels, pods, pomace, molasses, shells, and oilseed processing waste. Underutilized food byproducts, being rich in proteins, lipids, bioactive compounds, dietary fibers, and micronutrients, have attracted the interest of researchers, policymakers, and industry professionals for their potential to be valorized into value-added products [76,77].

Food-processing byproducts present a promising opportunity for producing sustainable nanoporous materials like cellulose aerogels. Valorizing these byproducts not only mitigates waste and environmental pollution but also generates economic value [7,78]. The fundamental approach involves extracting lignocellulosic content from the byproducts, which serve as a precursor for cellulose aerogel fabrication [7]. Moreover, food processing byproducts, being non-wood sources, have additional benefits compared to wood sources for cellulose extraction, including lower costs, easier processing, and lesser chemical and energy requirements, primarily due to their lower lignin content compared to wood [79]. However, the variable composition of different food processing byproducts can lead to inconsistent cellulose yield and aerogel performance, presenting significant challenges for standardization and large-scale production.

Table 1 summarizes the recent studies that utilized food processing byproducts to prepare cellulose aerogels with their preparation methods and potential applications. For instance, a study reported transforming spent ground coffee and apple pomace into high-value cellulose aerogels. These byproducts are usually discarded in landfills, generating environmental concerns. These lignocellulosic materials were processed into cellulose aerogels via the sol–gel method, followed by SC-CO_2_ drying. The resulting aerogels exhibited remarkable properties, with a specific surface area of 229 m^2^/g for coffee-derived cellulose aerogels and 208 m^2^/g for apple pomace-derived cellulose aerogels. Moreover, the apple pomace-derived cellulose aerogels (0.016 g/cm^3^) had a lower density compared to that of spent ground coffee aerogels (0.191 g/cm^3^) [7]. This suggests the importance of precursor selection that influences the structure and performance of aerogels.

Similarly, studies have reported the fabrication of cellulose aerogels from *Arundo donax* waste [4], hemp stems [8], pomelo peels [80], *Posidonia oceanica* waste [81], grape stalks [3], barley [48], wheat straw [82], and hemp stalks [83], where the fabricated aerogels showed notable properties with various applications, including absorption [80,84], food packaging [4,81], wastewater treatment [64], thermal and acoustic insulation [84], and controlled release of fertilizers [83] (Table 2). For example, a study demonstrated the application of cellulose aerogel derived from grape stalks for intelligent meat packaging [3]. When combined with Salep co-polymer and red grape anthocyanins, the resulting aerogel was lightweight and exhibited a highly porous framework with an elevated average pore size. These aerogels served as freshness indicators for minced meat, transforming color in response to acidic and alkaline environments [3]. In another study, cellulose aerogels synthesized from coir fibers demonstrated exceptional potential for sorption applications such as liquid spills, dye removal, and thermal insulation. The cellulose aerogels displayed water and oil sorption capacities of 22 and 16 g/g aerogel, respectively, with density and porosity of 0.0375 g/cm^3^ and 99.6%, respectively. Additionally, the aerogels exhibited an impressive methylene blue dye absorption capacity of up to 62 g/g, exceeding the adsorption capability of other natural material-derived adsorbents by nearly 100 times [85]. These studies highlight the functional tunability of cellulose aerogels for applications in specific target areas; however, the reproducibility and economic feasibility of cellulose aerogels remain to be further investigated.

Additionally, a study reported the impact of various pretreatments of cellulose extraction, namely alkaline, dilute acid, and acidified glycerol pretreatments, on the properties of cellulose aerogels of barley straw [48]. The aerogel from barley straw cellulosic fibers with the highest cellulose content (80% from acidified glycerol) exhibited the largest surface area (49.5 m^2^/g), compared to the aerogel from fibers with the lowest lignin content (6% from alkaline) that exhibited lowest mass per unit volume (0.0274 g/cm^3^) and an exceptionally high porosity (98.17%) [48]. Additionally, aerogels with higher hemicellulose content and porosity showed enhanced methylene blue adsorption and pore-filling properties. All aerogels demonstrated the potential for oil spillage cleanup and dye removal [48]. These findings emphasize the role of pretreatment chemistry in controlling both porosity and adsorption behavior of cellulose aerogels. However, pretreatments are often associated with high processing costs; therefore, achieving an optimal balance between cellulose yield, pretreatment efficiency, and processing cost remains a critical bottleneck requiring further research.

Different food processing byproducts exhibit varying chemical compositions and fiber structure that significantly influence the final cellulose yield [7,40,44,45,46,47,48,86], aerogel properties [7,87], and scalability of aerogels. For instance, corncob has been reported to yield a higher amount of cellulose compared to rice straw, pineapple leaf, and pineapple peel, primarily due to differences in chemical compositions among these byproducts [86]. Moreover, substantial variations in aerogel density have been reported, depending on the cellulose source. The density of cellulose aerogels extracted from apple pomace (0.016 g cm^−3^) was lower compared to that from spent ground coffee (0.19 g cm^−3^), which can be attributed to differences in the physicochemical characteristics of the respective biomass precursors of the byproducts [7]. Although food byproducts represent low-cost and sustainable sources for the synthesis of cellulose aerogels, studies evaluating optimal feedstocks for scalable synthesis remain limited [8,88]. The scalability of cellulose aerogels is influenced by several factors, including the availability and nature of the precursor, production process and type and amount of chemicals used. One of the studies reported the potential of sugarcane bagasse as a promising candidate for producing scalable nanocellulose aerogels, attributable to its widespread availability and suitability for scalable production processes [88]. Therefore, further research must evaluate the relationship between the aerogel performance and production cost to guide the translation of aerogels into the industries.

**Table 2:** Utilization of food processing byproducts to prepare cellulose aerogels and cryogels: preparation methods, properties, and potential applications.

Source	Cellulose Extraction Method	Aerogel/Cryogel Preparation Method	Physical Properties	Potential/Target Application	Reference
Spent ground coffee	-	Hot water rinsing → Ethanol/water solvent exchange → SC-CO_2_ drying	Specific surface area: 229 ± 20 m^2^/g Density: 0.191 ± 0.004 g/cm^3^ Water absorption: 13.4 ± 0.9%	Wastewater treatment, food packaging and thermal insulation	[7]
Apple pomace	-	Anhydrous ethanol mixing → Ethanol/water solvent exchange → SC-CO_2_ drying	Specific surface area: 208 ± 20 m^2^/g Density: 0.016 ± 0.001 g/cm^3^ Water absorption: 15.6 ± 0.4%	Potential application in aq. media	[7]
*Arundo donax* waste (leaves and stems)	Soxhlet treatment → NaClO_2_ → KOH treatment	Hot water extraction and ultrasound assisted treatment → Freeze drying	Density: 10.21–25.55 mg/cm^3^ Water vapor sorption: 0.39–1.09 g/g aerogel	Superabsorbent pads to preserve meat quality	[4]
Hemp stems	Soxhlet treatment → NaClO_2_ → KOH treatment	Citric acid gelation → Absolute ethanol solvent exchange → SC-CO_2_ drying	T_onset_ = 317 °C; T_max_ = 416 °C Surface area: 295.13 m^2^/g Average pore diameter: 7.17 nm Average pore width: 14.02 nm Density: 5.0 × 10^−4^ ± 7.0 × 10^−5^ g/cm^3^ Compressive strength: 40.01 kPa at 50% compressive deformation Water vapor sorption: 1.86 ± 0.10 g/g aerogel	Food packaging	[8]
Pomelo peels	NaOH → Sodium acetate buffer and NaClO_2_ → H_2_SO_4_	Cellulose + graphene oxide → Gelatinization → Lypholization	Density: 0.018 g/cm^3^ Porosity: 96% Surface area: 74 m^2^/g Pore size: 1.8–4 nm	Organic dye removal (Waste-water treatment)	[80]
*Posidonia oceanica* waste	Soxhlet treatment → NaClO_2_ → KOH	Cellulose → H_2_SO_4_ → Homogenization → Freeze drying	Density: 13–56 mg/cm^3^ Water sorption capacity: Up to ~1500% Oil sorption capacity: Up to ~1900%	Bioactive adsorbing pads in packaged fresh foods and preserving the quality of red meat	[81]
Grape stalks	Soxhlet treatment → NaClO_2_ → KOH	Cellulose + Salep solution → Homogenization → Freeze drying	Density: 14–15 mg/cm^3^ 90–92% porosity Water vapor sorption capacity: 0.4–0.6 g/g aerogel	Intelligent Meat packaging	[3]
Coir fibers	NaOH treatment	Fiber pulp → NaOH/Urea solution → Gelation → Ethanol solvent exchange → Freeze drying	Water absorption capacity: 22 g/g aerogel Density (lowest): 0.0375 g/cm^3^ Porosity (highest): 97.6% Oil absorption capacity: 16 g/g aerogel Dye absorption capacity: 62 g/g aerogel	Sorption application (Absorbent for liquid spill and dye removal), Thermal insulation	[85]
Sugarcane bagasse	NaOH → H_2_O_2_	Pulp + polyvinyl alcohol solution → Magnetic Stirring → Glyoxal → Homogenization → Freeze drying	Density: 0.012–0.108 g/cm^3^ Porosity: 93–99% Surface area: 309–368 m^2^/g Pore diameter (maximum): 11.3 nm Thermal conductivity: 0.026–0.044 W/mK Maximum sound absorption co-efficient: 0.861 Oil sorption capacity: 17–23 g/g aerogel Maximum compressive strength: 41 kPa	Thermal, acoustic insulator, oil adsorbent	[84]
Barley straw	NaOH → Hot water Dil. H_2_SO_4_ → Hot water Acidified glycerol → Hot water	Pulp + polyvinyl alcohol solution → Mixing → Sonication → Freeze drying	Density: 0.027–0.034 g/cm^3^ Porosity > 97% Oil adsorption capacity: 25–30 g/g aerogel Methylene blue adsorption capacity (highest): 10.65 mg/g Surface area: 24–50 m^2^/g	Oil spillage cleanup, dye removal	[48]
Hemp stalk	H_2_SO_4_ dipping → Steam explosion in NaOH → H_2_O_2_ bleaching → HCl hydrolysis → Homogenization	Carboxymethyl cellulose solution + citric acid → Gelation → Deionized water washing → Freeze drying → ADP and urea encapsulation	Water absorption capacity: 80 g/g aerogel Release percentage of nutrients in water: 44–48% Release percentage of nutrients in soil: 57–62%	Controlled release of fertilizers	[83]
Wheat straw	Soxhlet Extraction → NaClO_2_ and glacial acetic acid → NaOH → HCl	Cellulose pulp + NaOH/PEG → HCl + distilled water + butanol → Freeze drying	Density: 45–148 mg/cm^3^ Specific surface area: 47–101 m^2^/g Mean pore diameter: 17–18 nm Pore volume: 0.2–0.5 cm^3^/g	Oil and dye absorbents	[82]
Lupin hulls	Supercritical water treatment → NaClO_2_	Cellulose fibers → Ultrasonication → SC-CO_2_ drying/Freeze drying	Porosity: 96.6–99.4% Density: 0.009–0.05 g/cm^3^ Specific surface area: 16–115 m^2^/g	Food packaging, tissue engineering scaffolds	[62]
Sugarcane bagasse	NaOH → NaClO_2_ + glacial acetic acid	Pulp → H_2_SO_4_ → Ultrasonication → Sugarcane bagasse cellulose nanocrystals + chitosan from crustacean shells → Homogenization → Freeze drying	Specific surface area: 1.26–2.15 m^2^/g CO_2_ uptake: 5.78 mg/g at 273 K and 2.82 mg/g at 298 K	Sustainable CO_2_ capture	[89]
Pineapple peel	Fermentation by *Acetobacter xylinum* under aerobic conditions → NaOH	Cellulose fibers + water → Grinding → Freeze drying	Density: 0.0051 g/cm^3^ Specific surface area: 35 m^2^/g Cationic dye adsorption capacity: 28–49 mg/g	Wastewater treatment	[90]
Pineapple fibers	-	Blending → Polyvinyl alcohol crosslinking → Pre-freezing → Freeze drying	Porosity: 97–99% Density: 0.013–0.003 g/cm^3^ Thermal conductivity: 0.030–0.034 W/mK Noise reduction coefficient: 0.52 Compressive modulus: 1.64–5.34 kPa	Thermal and acoustic insulation	[91]

## 5. Environmental and Economic Benefits of Utilizing Food Processing Byproducts for Producing Cellulose Aerogels

The utilization of food processing byproducts for the preparation of cellulose aerogels for diverse applications is a sustainable approach to reducing negative environmental impact, further improving resource efficiency, and lowering the cost of waste disposal. This approach can help valorize processing waste into producing high-value products that could be utilized for diverse applications [7,8]. The valorization of food processing byproducts contributes to mitigating greenhouse gas emissions, improving ecosystem services, and supporting sustainable resource management policies. The environmental benefit of producing high-value materials from food processing byproducts ultimately depends on the production method [92]. Therefore, there is a critical need for life cycle assessments and technoeconomic analyses to develop a holistic approach for upcycling these food processing byproducts. In particular, the chemicals used in cellulose extraction need to be considered critically.

Food-processing byproducts are often low-cost and readily available materials, making them an attractive, cost-effective raw material for aerogel synthesis and suitable for large-scale use. Moreover, transforming food processing byproducts will also generate new market opportunities and promote job growth in areas with agricultural and food processing industries. Thus, integrating food byproduct valorization in pre-existing industries supports a more sustainable and robust economy [89]. Additionally, the key to achieving economic benefits depends on the production process, quality, and applications of the resulting products achieved from food processing byproducts [92].

Additionally, food processing byproducts contain hemicellulose as well as lignin, in addition to cellulose, which are usually discarded during cellulose extraction [38]. The valorization of these underutilized biopolymers can further enhance environmental and economic sustainability, reinforcing the principle of a circular economy. Lignin waste can be valorized into biofuels or bioenergy, or it can be converted into bioplastics, composites, or chemicals like phenols, aldehydes, xylitol, and saccharides [38,93]. Similarly, hemicellulose, an underexploited side stream component of food byproducts, can be fractionated and fine-tuned for applications in water-sensitive materials, prebiotic oligosaccharides, active food packaging, and biomedical devices [38].

Despite these advantages, several critical barriers hinder the large-scale production of cellulose aerogels. These involve the synthesis of aerogels via energy and cost-intensive processing methods, particularly drying techniques [94]. The production processes are time-consuming, leading to increased operational costs, limiting the adoption of cellulose aerogels at an industrial scale [94,95,96]. Additional challenges involve variation in composition, quantity and quality of food byproducts, fluctuations in availability and supply attributable to the geographical region or climate variation, and regulatory aspects [97]. These inconsistencies may result in batch-to-batch variations impacting reproducibility and variability. Therefore, these challenges need to be addressed by incorporating low-energy and economically viable processing methods, pretreatment standardization, and robust quality control strategies.

To address these challenges, ongoing research has focused on optimizing the synthesis process of cellulose aerogels to reduce energy consumption, improve scalability, and reduce environmental and economic impact [69,95]. As SC-CO_2_ drying is a major drying process involved in the preparation of cellulose aerogels, efforts are being made to optimize the drying process to reduce the environmental impact and production cost [6,66,69,73]. This mainly involves lowering drying time, reducing energy consumption as well as CO_2_ consumption by improving mass transfer conditions and recycling. For instance, a study reported that maintaining a Biot number above 65 within specific pressure (9–17 MPa) and temperature (313–323 K) ranges has been shown to accelerate drying and reduce solvent use by promoting a diffusion-limited regime [69]. Another study explored a foam template-assisted (FTA) strategy to enable atmospheric pressure drying of nanocellulose aerogels, offering a low-cost and structurally robust alternative to conventional methods [97]. Additionally, continuous manufacturing and integrated processing, combined with alternative techniques such as 3D printing, were recommended to significantly reduce aerogel production costs and enhance material functionality [98]. These advancements further highlight the potential for developing sustainable and economically viable cellulose aerogels from food processing byproducts, thus strengthening their potential for broader industrial adoption.

## 6. Conclusions and Future Perspectives

The environmental concerns associated with the use of fossil resources to fulfill the demands of the increasing population have increased the interest in producing low-cost, sustainable, biodegradable materials. Cellulose aerogels are gaining importance due to their exceptional properties, demonstrating promising applications in diverse fields, including oil adsorbents, wastewater treatment, drug delivery, and food packaging. Although there are numerous raw materials to produce cellulose aerogels, the utilization of food processing byproducts for cellulose aerogel production presents a sustainable approach to producing high-value products, thus aligning with the principles of circular bioeconomy. The transformation of the byproducts into cellulose aerogels not only reduces environmental pollution and waste but also contributes to economic sustainability. Despite significant progress, there are challenges in optimizing the production process of cellulose aerogels due to the variable lignocellulosic compositions of the food processing byproducts. To address these obstacles, future studies should focus on the standardization of processing methods to improve scalability and batch-to-batch consistency. This can involve developing energy-efficient and eco-friendly cellulose aerogel production methods, such as using green solvents. Further, drying techniques, particularly SC-CO_2_ drying, freeze drying, and ambient pressure drying, must be optimized to ensure a reduction in energy consumption, drying time, and overall production cost. Hybrid drying techniques can be explored to further enhance sustainability and economic viability for cellulose aerogel preparation. Future studies should also focus on utilizing other components like lignin and hemicellulose, extracted during the production process, to make the approach more sustainable and follow the principles of circular bioeconomy. In addition, life cycle and technoeconomic assessments need to be conducted to better understand the advantages/disadvantages of upcycling food processing byproducts into cellulose aerogels. These tools can guide decision-making regarding the choice of solvents, optimal production processes, and strategies to reduce environmental and economic burden. A comparative life cycle and technoeconomic analysis of cellulose aerogel production from food byproducts and conventional feedstocks can offer valuable insights into the feasibility of large-scale industrial adoption. By focusing on these key areas, the scalability, cost-effectiveness, and environmental sustainability of cellulose aerogels from food processing byproducts can be improved, paving the way for their commercial implementation.

## Figures and Tables

**Table 1:** Cellulose, hemicellulose, and lignin contents of common food processing byproducts.

Food Processing Byproducts	Cellulose (%, *w*/*w*)	Hemicellulose (%, *w*/*w*)	Lignin (%, *w*/*w*)	Reference
Spent ground coffee	12	39	24	[44]
Apple pomace	36	11	19	[45]
Hemp stem	41	21	22	[8]
Grape stalks	30	21	17	[46]
Coir fibers	36–43	0.15–0.25	32–45	[47]
Sugarcane bagasse	44	28	21	[40]
Barley straw	37	34	28	[48]
Hemp stalk	46	24	23	[49]
Wheat straw	33	30	14	[40]
Pineapple peels	24	30	6	[50]

## Data Availability

No new data were created or analyzed in this study. Data sharing is not applicable to this article.
